# GIW and InCoB, two premier bioinformatics conferences in Asia with a combined 40 years of history

**DOI:** 10.1186/1471-2164-16-S12-I1

**Published:** 2015-12-09

**Authors:** Christian Schönbach, Paul Horton, Siu-Ming Yiu, Tin Wee Tan, Shoba Ranganathan

**Affiliations:** 1 Department of Biology, School of Science and Technology, Nazarbayev University, Astana 010000, Republic of Kazakhstan; 2 Center for AIDS Research and International Research Center for Medical Sciences, Kumamoto University, Kumamoto 860-0811, Japan; 3 Computational Biology Research Center, National Institute of Advanced Industrial Science and Technology, Tokyo 135-0064, Japan; 4 Department of Computational Biology, Graduate School of Frontier Sciences, University of Tokyo, Japan; 5 Department of Computer Science, Faculty of Engineering, The University of Hong Kong, Hong Kong, HKSAR; 6 Department of Biochemistry, Yong Loo Lin School of Medicine, National University of Singapore, Singapore 117599; 7 Department of Chemistry and Biomolecular Sciences, Macquarie University, Sydney NSW 2109, Australia

## Abstract

Knowledge discovery in bioinformatics thrives on joint and inclusive efforts of stakeholders. Similarly, knowledge dissemination is expected to be more effective and scalable through joint efforts. Therefore, the International Conference on Bioinformatics (InCoB) and the International Conference on Genome Informatics (GIW) were organized as a joint conference for the first time in 13 years of coexistence. The Asia-Pacific Bioinformatics Network (APBioNet) and the Japanese Society for Bioinformatics (JSBi) collaborated to host GIW/InCoB2015 in Tokyo, September 9-11, 2015. The joint endeavour yielded 51 research articles published in seven journals, 78 poster and 89 oral presentations, showcasing bioinformatics research in the Asia-Pacific region. Encouraged by the results and reduced organizational overheads, APBioNet will collaborate with other bioinformatics societies in organizing co-located bioinformatics research and training meetings in the future. InCoB2016 will be hosted in Singapore, September 21-23, 2016.

## Introduction

For the first time the 26th International Conference on Genome Informatics (GIW) and the 14th International Conference on Bioinformatics (InCoB) were jointly held in Tokyo, September 9-11, 2015 [1]. Three satellite workshops on BioConductor, genome privacy, PRIVAGEN 2015 and the Bioconductor Asia-Pacific Developer Meeting preceded GIW/InCoB2015. The conference was attended by approximately 200 delegates with two-thirds coming from Taiwan and Japan and a third mostly from China, USA, India, Korea, Malaysia, Singapore and France. Up to four parallel sessions provided sufficient time for 89 oral and 78 poster presentations and six keynote talks covering progress and remaining challenges in genomics, proteomics and disease informatics. Gil Ast presented how chromatin organization, epigenetics and alternative splicing work together while Yana Bromberg focussed on interpreting genomic data to inform pathogenesis pathways. Edward Marcotte connected the proteome and human disease via evolutionary links while Mark S. Baker, the current President of the HuPO (the Human Proteome Organization) expounded the Human Proteome Project's efforts to find the "missing proteins" and develop a common informatics language. Arne Elofsson detailed what is missing from a complete structural map of the cell while and Annie de Groot revealed a computational pipeline for personalized cancer vaccines.

## Manuscript submission and review

GIW/InCoB2015 offered authors of original research papers a choice of seven journal tracks for submission: BMC Genomics, BMC Bioinformatics, BMC Systems Biology, BMC Medical Genomics, Bioinformatics, IEEE/ACM Transactions on Computational Biology, Bioinformatics, and the Journal of Bioinformatics and Computational Biology. Of 106 manuscript submissions, 51 (48%) were accepted after a cycle of peer reviews and one or two revisions by at least two members of the 110 members of the Program Committee and 39 external sub-reviewers (Additional file 1). Manuscripts with top reviewer scores were select for the Best Paper Awards. The five award-winning manuscripts in the BMC and Bioinformatics tracks and their authors are listed in Additional file 2.

The GIW/InCoB2015 BMC Genomics supplement [2] includes 15 papers. Another six biomedical genomics-related papers that are covered in this introduction are published in the BMC Medical Genomics supplement [3]. The remaining 16, mostly tool/software and six biological networks and six systems-related manuscripts are published in BMC Bioinformatics [4] and BMC Systems Biology supplements [5], respectively. Catering to requests of offering a more diverse range of journals we collaborated with Bioinformatics [6], IEEE/ACM Transactions on Computational Biology and Bioinformatics [7], and the Journal Bioinformatics and Computational Biology [8]. In total, ten manuscripts have been published in these journals.

## Disease informatics

Four papers represent a cross section of topics relevant in disease informatics that ranged from investigating epigenetic contributions in thyroid cancer etiology by analysing gene expression and methylation profiles [9] to integrated pharmacological profiling and biological network based drug target discovery. Ryall *et al*. [10] predicted compounds that may inhibit kinases in triple negative breast cancer cell line models. The targeted kinases were derived from integrated analysis of gene expression and pharmacological profiles supplemented with *in vitro *kinase binding assays data. The reduced probability of misinterpreted or false kinase dependencies and applicability of the kinase addition ranker tool to patient samples for improving drug therapies highlight the power of bioinformatics in translational research. Disease network studies are also gaining importance in rational drug discovery as demonstrated by Wong *et al*. [11]. Protein-protein interaction network analysis across bladder, colorectal and liver lung combined computer-aided drug design with yielded core network proteins that represent multiple drug targets for each cancer. Handen and Ganapathiraju [12] took the disease network approach to the next level by gleaning information from relations of genes across different diseases types for example between an immune response to infectious diseases and autism or schizophrenia. Their inter-disease enrichment and network analysis of human proteins was enabled by the web-based tool LENS [12].

## Immunoinformatics

The first step in rational vaccine design is antigen selection. Computer-aided T and B cell epitope predictions can save time and cost. Yet escape mutations, antigenic drift, emerging new viral strains and desired broad cross-reactivity are recurring issues in all these efforts.

The new enterovirus genotyping tool EVIDENCE [13] allows users to detect recombinants and potential new strains. Sun and Brusic [14] analyzed discontinuous peptides and their neutralizing activity across HCV strains. The resulting cataloging method has the potential to be used in the informed knowledge-driven selection of HCV strains for the development of neutralizing antibody tests. Huang *et al*. [15] took a more technical approach limited to HCV linear B cell epitopes that resulted interpretable rules mining system for epitope prediction. A new integrated tool that enhances the identification of potential T-cell epitopes by combining immunogenic peptide prediction with visualization of conserved peptide sequence blocks was developed Olson *et al*. [16]. Siaugi *et al*. [17] integrated influenza protein sequences and metadata into g-FLUA2H database to provide a resource for better understanding of mutation transmission dynamics between hosts.

Several miRNAs have been reported in context of tolerance to endotoxins [18]. Chiu and Wu [19] analyzed miRNA profiles derived from mouse bone marrow-derived macrophages and identified 431 miRNAs and 498 differentially expressed target genes that await further functional characterization.

## Genomics

Estimates of recombination rates are mostly based on linkage disequilibrium calculations on the population level. Chen *et al*. [20] developed the tool ARG-walker that uses a graph mining algorithm to estimate the odds of individual-specific recombination from random walks on the ancestral recombination graphs. Screening of HapMap [21] data revealed new cis- and trans-regulatory candidates of recombination hotspots.

Improved next generation sequencing technology led to an increased depth and quality of reads. Fang *et al*. [22] investigated the potential of subset selection of reads or read pairs in genome assembly without compromising the quality of the assembly. Initial results are promising the method of subset selection and usability awaits further improvements.

PCR-based large-scale DNA fingerprinting and genotyping are frequently used in plant breeding. The mostly automated procedure involves the processing of gel electrophoresis images. Curved lanes and suboptimal exposure are known to interfere with the automatic image analysis. Intarapanich *et al*. [23] implemented a new lane segmentation algorithm, GELect in Java with imageJ library that can handle even highly curved lanes correctly and derive genotypes by grouping similar banding patterns.

## Epigenomics

Lee *et al*. [24] compared and integrated mapping results of three methylation detection tools based on average detected methylation levels, read depth and a weighted probability measure. The integrative approach resulted in improved accuracy and sensitivity of methylation detection. Differentially methylated regions (DMR) may represent known transcription factor binding sites or new candidate regulatory elements. The developers of Bisulfighter [25] improved the DMR detection capability from bisulfite sequencing data by replacing empirical parameters with emission functions for HMM-based DMR detection [26] Complimentary to the aforementioned bisulfite sequencing primary data analysis tools is MethGO [27]. This analysis tool for bisulfite sequence alignments provides users options to see the methylation site coverage distribution, methylation states at defined regions (e.g. promoter and transcription factor (TF) binding sites) and calling functions for copy number variations (CNV) and single-nucleotide polymorphisms (SNPs).

## Gene and transcription networks

The cooperation among TFs is an important feature in transcriptional regulation and network construction efforts utilizing data on TF pairs data. Most existing cooperative TF pairs prediction algorithms are overly reliant on improved cooperative measures. Wu and Lai [28] developed an algorithm that considers integrated yeast TF binding and perturbation data and outperformed twelve other algorithms. Interestingly, their benchmarking results also indicate that the process of defining biologically plausible targets of a TF might be more important than optimizing cooperative measures.

Gene expression time-course data are often used to infer gene interaction networks. The ability of tools to process only single rather than multiple data sets limits the ability to generate reliable time-lagged gene interactions. Liu *et al*. [29] developed an algorithm that detects conserved sub-sequential patterns of gene expression from multiple data sets which bypasses the limitations of local and global proximity measure methods.

## Functional and structural genomics

Zinc finger transcription factor 268 (Zif268 also known as Egr-1, NGFI-A, Krox-24, TIS8 or ZENK) is involved in neuronal plasticity [30]. Dutta *et al*. [31] used its structure to investigate dynamics of water molecules on the binding affinity of the zinc finger protein to DNA. Hydrogen bond retention, desolvation and DNA deformation were found to affect the affinity to target DNA sites. Although the results are case-based they have broader implications on both prediction accuracy of structure-based TF binding affinity predictions and interpretation of biological functions due to binding affinity differences.

Membrane transporters are in important class of proteins in drug target design. Since the number of resolved 3D structures is limited the high accuracy prediction of membrane transporters from primary sequence data is of considerable interest. Liou *et al*. [32] applied dipeptide and amino acid propensity scoring to develop a scoring card-based support vector machine tool for analyzing structure and physicochemical properties of membrane transporters.

Low resolution mass spectrometry data derived from metabolome or proteome studies often show overlapping peak distributions. Wijetunge *et al*. [33] implemented in MATLAB^® ^an algorithm for the improved peak detection and parameter estimation to increase the accuracy of molecule identification.

## Conclusion

Joint or co-located meetings offer participants a diverse program at lower cost than separate events and provide a unique networking opportunity. Therefore, InCoB will partner different stakeholders in the bioinformatics community in the future. InCoB2016 [34] will be held in Singapore from Sept. 21-23, 2016.

## List of abbreviations

AIST - National Institute of Advanced Industrial Science and Technology, Japan

APBioNet- Asia-Pacific Bioinformatics Network

IEEE/ACM IEEE Communications Society/Association for Computing Machinery

GIW - International Conference on Genome Informatics

GOBLET - Global Organisation for Bioinformatics Learning, Education and Training

HMM - Hidden Markov Model

InCoB - International Conference on Bioinformatics

JSBi - Japanese Society for Bioinformatics

TF - transcription factor

## Competing interests

The authors declare that they have no competing interests.

## Authors' contributions

CS and SR wrote the introduction. CS and SMY (Program Committee Co-chairs) managed the review, revision and decision processes. TWT, PH and SR supported the post-acceptance and editorial processing, respectively. All authors have read and approved the final manuscript.

## Supplementary Material

Additional File 1 **List of GIW/InCoB2015 Reviewers**. (*.pdf) Click here for file

Additional File 2 **GIW/InCoB2015 Best Paper Awards**. (*.pdf) Click here for file
