# Supplementary material for: GIW and InCoB, two premier bioinformatics conferences in Asia with a combined 40 years of history
Source: BMC Genomics. 2015 Dec 9;16(Suppl 12):I1. doi: 10.1186/1471-2164-16-S12-I1 (PMC4682400; doi:10.1186/1471-2164-16-S12-I1)
Supplement: Additional File 1 — List of GIW/InCoB2015 Reviewers. (*.pdf) [file 1471-2164-16-S12-I1-S1.pdf]

Additional File 1. List of GIW/InCoB2015 Reviewers

We are grateful to the members of GIW/InCoB2015 Scientific Program Committee and the sub-reviewers (listed alphabetically below) for peer reviewing manuscripts submitted to the GIW/InCoB2015 supplement issues of BMC Genomics, BMC Medical Genomics, BMC Bioinformatics or BMC Systems Biology.

GIW/InCoB2015 Scientific Program Committee:

1. Shandar Ahmad, National Institute of Biomedical Innovation, Japan
2. Tatsuya, Akutsu, Kyoto University, Japan
3. Shunsuke Aoki, Kyushu Institute of Technology, Japan
4. Masanori, Arita, National Institute of Genetics, Japan
5. Nicola Armstrong, Garvan Institute of Medical Research, Australia
6. Kiyoshi Asai, The University of Tokyo, Japan
7. Arsen Batagov, A*STAR, Bioinformatics Institute, Singapore
8. Alex Bateman, European Bioinformatics Institute, UK
9. Abdul Baten, Southern Cross University, Australia
10. Michael Beer, Johns Hopkins University, USA
11. Mikael Boden, The University of Queensland, Australia
12. Edmond Breen, APAF, Ltd. Australia
13. Vladimir Brusic, Nazarbayev University, Kazakhstan
14. Yiyu Cai, Nanyang Technological University, Singapore
15. Zhi-Wei Cao, Shanghai Center for Bioinformatics Information Technology, PR China
16. Filippo Castiglione, National Research Council of Italy, Institute for Computing Application, Italy
17. Jonathan Chan, King Mongkut's University of Technology Thonburi, Thailand
18. Kun-Mao Chao, National Taiwan University, Taiwan
19. Michael Charleston, The University of Sydney, Australia
20. Ming Chen, Zhejiang University, PR China
21. Xin Chen, Zhejiang University, PR China
22. Yi-Ping Phoebe Chen, La Trobe University, Australia
23. Brian Chen, Lehigh University, USA
24. Mohd Firdaus-Raih, Universiti Kebangsaan Malaysia, Malaysisa
25. Anne Fischer, International Centre of Insect Physiology and Ecology, Kenya
26. Andrew French, University of Nottingham, UK
27. Matthias Futschik, Universidade do Algarve, Portugal
28. Bruno Gaeta, University of New South Wales, Australia
29. Ge Gao, Peking University, PR China
30. Pascale Gaudet, Swiss Institute of Bioinformatics, Switzerland
31. Charles Gilman, Nazarbayev University, Kazakhstan
32. Michael Gromiha, Indian Institute of Technology Madras, India
33. Iman Hajirasouliha, Stanford University, USA
34. Wing-Kai Hon, National Tsing Hua University, Taiwan
35. Paul Horton, AIST Computational Biology Research Center, Japan

36. Chia-Lang Hsu, National Taiwan University, Taiwan
37. Wenlian Hsu, Academia Sinica, Taiwan
38. Chun-Hsi Huang, University of Connecticut, USA
39. Hsuan-Cheng Huang, National Yang-Ming University, Taiwan
40. Ming-Jing Hwang, Academia Sinica, Taiwan
41. Asif M. Khan, Perdana University, Malaysia
42. Javed Khan, Harry Perkins Institute for Medical Research, Australia
43. Tsung Fei Khang, University of Malaya, Malaysia
44. Daisuke Kiga, Tokyo Institute of Technology, Japan
45. Sun Kim, Seoul National University, RO Korea
46. Akira Kinjo, Osaka University, Japan
47. Kengo Kinoshita, Tohoku University, Japan
48. Tetsuya Kobayashi, The University of Tokyo, Japan
49. Akihiko Konagaya, Tokyo Institute of Technology, Japan
50. Shinji Kondo, National Institute of Polar Research, Japan
51. Anton Kratz, RIKEN Center for Life Science Technologies, Japan
52. Gaurav Kumar, Virginia Commonwealth University, USA
53. Manish Kumar, University of Delhi, India
54. Hiroyuki Kurata, Kyushu Institute of Technology, Japan
55. Igor V. Kurochkin, A*STAR, Bioinformatics Institute, Singapore
56. Chee Keong Kwoh, Nanyang Technological University, Singapore
57. Chih Lee, Illumina Inc., USA
58. Hyunju Lee, Gwangju Institute of Science and Technology, RO Korea
59. Henry CM. Leung, The University of Hong Kong, Hong Kong
60. Guo-Zheng Li, Tongji University, PR China
61. Jinyan Li, University of Technology Sydney, Australia
62. Xiaoli Li, A*STAR, Institute for Infocomm Research, Singapore
63. Jie Li, Harbin institute of Technology, PR China
64. Wei Lin, Fudan University, PR China
65. Bin Liu, Harbin institute of Technology, PR China
66. Bo Liu, Harbin institute of Technology, PR China
67. Adeel Malik, Perdana University, Malaysia
68. Hiroshi Mamitsuka, Kyoto University, Japan
69. Hideo Matsuda, Osaka University, Japan
70. Bui Quang Minh, Max F. Perutz Laboratories, Austria
71. Lenny Moise, University of Rhode Island, USA
72. Santo Motta, University of Catania, Italy
73. Kenta Nakai, The University of Tokyo, Japan
74. See-Kiong Ng, Institute for Infocomm Research
75. Ka-Lok Ng, Department of Biomedical Informatics, Asia University
76. Lars Rønn Olsen, Dana-Farber Cancer Institute, USA
77. Ashwini Patil, The University of Tokyo, Japan
78. Shoba Ranganathan, Macquarie University, Australia
79. Yasubumi, Sakakibara, Keio University, Japan
80. Daniele Santoni, NRC Italy, Institute for System Analysis and Computer Science "Antonio Ruberti", Italy

81. Christian Schönbach, Nazarbayev University, Kazakhstan
82. Jun Sese, AIST Computational Biology Research Center, Japan
83. Hong-Bin Shen, Shanghai Jiaotong University, PR China
84. Tetsuo Shibuya, The University of Tokyo, Japan
85. Daron Standley, Osaka University, Japan
86. Chinh Tran-To Su, Nanyang Technological University, Singapore
87. Yanni Sun, Michigan State University, USA
88. Durai Sundar, Indian Institute of Technology Delhi, India
89. Wing-Kin Sung, National University of Singapore, Singapore
90. Sing-Hoi Sze, Texas A&M University, USA
91. Yoshihiro Taguchi, Chuo University, Japan
92. Yoichi Takenaka, Osaka University, Japan
93. Martti Tammi, Sime Darby Technology Centre Sdn Bhd, Malaysia
94. Paolo Tieri, National Research Council of Italy, Institute for Applied Mathematics "Mauro Picone", Italy
95. Joo Chuan Tong, A*STAR, Institute of High Performance Computing, Singapore
96. Sissades Tongsima, National Center for Genetic Engineering and Biotechnology, Thailand
97. Ikuo Uchiyama, National Institute for Basic Biology, Japan
98. Mauno Vihinen, Lund University, Sweden
99. Guohua Wang, Harbin institute of Technology, PR China
100. Dongqing Wei, Shanghai Jiaotong University, PR China
101. Limsoon Wong, National University of Singapore, Singapore
102. Thomas Wong, The University of Hong Kong, Hong Kong
103. Yufeng Wu, University of Connecticut, USA
104. Yingqiu Xie, Nazarbayev University, Kazakhstan
105. Wanling Yang, The University of Hong Kong, Hong Kong
106. Kevin Yip, The Chinese University of Hong Kong, Hong Kong
107. Siu-Ming Yiu, The University of Hong Kong, Hong Kong
108. Guang Lan Zhang, Boston University, USA
109. Liqing Zhang, Virginia Polytechnic Institute and State University, USA
110. Shanfeng Zhu, Fudan University, PR China.

Sub-reviewers (39):

Abdul Rahim Ahmad, Tanvir Alam, Costas Bouyioukos, Hwann-Tzong Chen, Chong Chu, Larry Croft, Yongchao Dou, Isabel Duarte, Christine Eng, Worrawat Engchuan, Fereydoun Hormozdiari, Kendrick Hougen, Yongli Hu, Lin Huan, Salvatore Ingrassia, Seppo Karrila, Joaquin Lamia, Xin Li, Lloyd Low, Nikolaos Papanikolaou, Jinwoo Park, Youngjune Park, Ralph Patrick, José Pedro Pinto, Victoria Popic, Setia Pramana, Elisabet Pujadas, Govindarajan Kunde Ramamoorthy, Sungmin Rhee, Edward Shih, Vasanth Singan, Prashanth Suravajhala, C. Conover Talbot Jr, Bimo Tejo, Songsak Tongchusak, Greg Tucker-Kellogg, Min Wu, Chao Xie and Junjie Zhu.
