# Supplementary material for: GIW and InCoB, two premier bioinformatics conferences in Asia with a combined 40 years of history
Source: BMC Genomics. 2015 Dec 9;16(Suppl 12):I1. doi: 10.1186/1471-2164-16-S12-I1 (PMC4682400; doi:10.1186/1471-2164-16-S12-I1)
Supplement: Additional File 2 — GIW/InCoB2015 Best Paper Awards. (*.pdf) [file 1471-2164-16-S12-I1-S2.pdf]

Additional File 2. GIW/InCoB2015 Best Paper Awards

BMC Genomics GIW/InCoB2015 Supplement

Chiu C-C, Wu W-S: **Investigation of microRNAs in mouse macrophage responses to lipopolysaccharide-stimulation by combining gene expression with microRNA-target information.** *BMC Genomics* 2015, 16(Suppl 12):S13.

BMC Medical Genomics GIW/InCoB2015 Supplement

Olsen LR, Simon C, Kudahl UJ, Bagger FO, Winther O, Reinherz EL, et al.: **A computational method for identification of vaccine targets from protein regions of conserved human leukocyte antigen binding**. *BMC Med Genomics* 2015, **8**(Suppl 4):S1.

BMC Bioinformatics GIW/InCoB2015 Supplement

Vandewater L, Brusic V, Wilson W, Macaulay L, Zhang P: **An adaptive genetic algorithm for selection of blood-based biomarkers for prediction of Alzheimer's disease progression.** *BMC Bioinformatics* 2015, **16**(Suppl 18):S1.

BMC Systems Biology GIW/InCoB2015 Supplement

Wong Y-H, Wu C-C, Lai H-Y, Jheng B-R, Weng H-Y, Chang T-H *et al.*: **Identification of network-based biomarkers of cardioembolic stroke using a systems biology approach with time series data**. *BMC Syst Biol* 2015, **9**(Suppl 6):S4.

Bioinformatics GIW/InCoB2015 article

Zhang Z, Wang J, Luo J, Ding X, Zhong J, Wang J, et al: **Sprites: detection of deletions from low-coverage sequencing data by re-aligning split reads**. *Bioinformatics* 2015, *accepted*.
